# Reducing depression during the menopausal transition: study protocol for a randomised controlled trial

**DOI:** 10.1186/1745-6215-15-312

**Published:** 2014-08-06

**Authors:** Osvaldo P Almeida, Kylie Marsh, Leon Flicker, Martha Hickey, Andrew Ford, Moira Sim

**Affiliations:** Western Australian Centre for Health & Ageing (M573), Centre for Medical Research of the Perkins Institute for Medical Research, University of Western Australia, 35 Stirling Highway, Crawley, WA 6009 Australia; School of Psychiatry & Clinical Neurosciences, University of Western Australia, 35 Stirling Highway, Crawley, WA 6009 Australia; Department of Psychiatry, Royal Perth Hospital, Perth, WA 6000 Australia; School of Medicine and Pharmacology, University of Western Australia, 35 Stirling Highway, Crawley, WA 6009 Australia; Department of Geriatric Medicine, Royal Perth Hospital, Perth, WA 6000 Australia; Department of Obstetrics & Gynaecology, University of Melbourne and Royal Women’s Hospital, Parkville, VIC 3052 Australia; School of Medical Sciences, Edith Cowan University, Joondalup, WA 6027 Australia

**Keywords:** perimenopause, menopausal transition, depression, stress, anxiety, health promotion, prevention, randomised controlled trial

## Abstract

**Background:**

The menopausal transition (MT) is a biological inevitability for all ageing women that can be associated with changes in mood, including depressive symptoms. There is tentative evidence that women who develop depression during the MT have greater risk of subsequent depressive episodes, as well as increased health morbidity and mortality. Thus, preventing depression during the MT could enhance both current and the future health and well-being of women. This study aims to test the efficacy of a client-centred health promotion intervention to decrease the 12-month incidence of clinically significant symptoms of depression among women undergoing the MT.

**Methods/Design:**

This randomised controlled trial will recruit 300 women undergoing the MT living in the Perth metropolitan area. They will be free of clinically significant symptoms of depression and of psychotic or bipolar disorders. Consenting participants will be stratified for the presence of subsyndromal symptoms of depression and then randomly assigned to the intervention or control group. The intervention will consist of eight telephone health promotion sessions that will provide training in problem solving and education about the MT, healthy ageing, depression and anxiety, and management of chronic health symptoms and problems. The primary outcome of interest is the onset of a major depressive episode according the DSM-IV-TR criteria during the 12-month follow-up or of clinically significant symptoms of depression, as established by a score of 15 or greater on the Patient Health Questionnaire (PHQ-9). Secondary outcomes of interest include changes in the severity of symptoms of depression and anxiety (Hospital Anxiety and Depression Scale, HADS), quality of life (Short Form Health Survey, SF-12), and lifestyle.

**Discussion:**

Current evidence shows that depressive symptoms and disorders are leading causes of disability worldwide, and that they are relatively common during the MT. This study will use a multifaceted health promotion intervention with the aim of preventing depression in these women. If successful, the results of this trial will have implications for the management of women undergoing the MT.

**Trial registration:**

Australian and New Zealand Clinical Trials Registry ACTRN12613000724774. Date registered: 1 July 2013.

## Background

Depression is a leading cause of disability and health-related costs in Australia and worldwide [1, 2], and affects between 4 and 14% of the population at any point in time [2]. The prevalence of depression is consistently higher in women than in men across the reproductive lifespan, with an increase in the prevalence of clinically significant depressive symptoms noted during the years that overlap with the menopausal transition (MT) [3–6]. The prospective midlife Study of Women’s Health Across the Nation (SWAN) in the United States indicated that nearly one in every four women experience clinically significant depressive symptoms at this time [7]. This, together with further confirmatory studies, led the National Institute of Aging to propose in 2010 that active screening of depressive symptoms should become an integral part of the assessment of all women going through the MT [8].

### What is the menopausal transition?

All ageing women with intact ovaries go through the MT, which is triggered by low ovarian follicular reserve that leads to elevated follicle-stimulation hormone, fluctuating oestradiol and reduced progesterone levels [9]. The MT commences at around age 47 and lasts 4 to 7 years [10]. The ‘menopause’ is the final menstrual period and occurs at a mean age of 51 years, and by age 55 nearly all women are postmenopausal [11]. Symptoms typically associated with the MT include irregular menstrual cycles, vasomotor symptoms (hot flushes and night sweats), sleep disturbances, vaginal dryness and dyspareunia, decreased libido, urinary symptoms, muscle and joint pains, and mood disturbance [10]. Importantly, typical symptoms associated with the MT (for example, hot flushes and night sweats) are amenable to change by means of biological or behavioural interventions [12, 13].

### Why is the prevalence of depressive symptoms high during the menopausal transition?

The reasons for the increased vulnerability to depression during the MT are not fully understood. Women with past history of depression are nearly five times more likely to have a recurrence of depressive illness during the menopausal transition, whereas women with no history of depression are two to four times more likely to report depressed mood compared with premenopausal women [6, 14, 15]. In some studies, the changing hormonal milieu has been associated with depressive symptoms during the MT [16, 17], but other factors may be as important, such as prolonged hot flushes, weight gain, comorbid diseases, poor social support, changing psychosocial roles, anxiety and perceived loss of control [18–21].

### Can we prevent depression during the menopausal transition?

Despite growing recognition that the MT is a period of high vulnerability to depression, no previous studies have attempted to prevent this happening. The spectrum of physiological, psychological and social changes that women undergoing the MT have to negotiate indicate that effective preventive strategies must be multifaceted (in the same way that strategies to reduce the prevalence of cardiovascular events have greater chance of success when multiple risk factors are targeted simultaneously, such as hypertension, diabetes, dyslipidaemia, physical activity, diet, alcohol use and smoking). Moreover, past history of depression increases the risk of postmenopausal depression, cardiovascular events, substance abuse and mortality [22, 23], the successful prevention and treatment of depression during the MT is expected to have long-term beneficial consequences for our ageing population. Clinical recommendations on managing the MT promote a broad approach to prevention and management of depression [24], including education, lifestyle changes (diet, exercise, smoking, and alcohol consumption), optimisation of general health and social support, minimisation of vasomotor symptoms, problem solving strategies and, if depression is prominent (that is, major depressive episode or dysthymia), antidepressant treatment and/or psychotherapy [25]. Oestrogen-containing hormone therapy is recommended for women with moderate to severe vasomotor symptoms without other contra-indications [13], and preliminary evidence suggests that it may also improve mood [16]. As the origin of depression during the MT is most likely multifactorial, its optimal management and prevention should target multiple factors, which is consistent with the results of successful interventions designed to decrease the prevalence of depression and suicide ideation in later life [26].

### Women undergoing the menopausal transition require a targeted health promotion intervention

The MT affects all ageing women with intact ovaries. Marked physiological and psychosocial changes characterise this unique period of a woman’s life, including symptoms such as hot flushes and night sweats, remodelling of body shape, shifting social roles (for example, children moving away from home), and onset of chronic illnesses (such as diabetes and hypertension). All these factors may interact to facilitate the development of depressive symptoms during the MT. As depression and poor lifestyle practices in mid life have been associated with poor health outcomes in older age [11, 27–29], developing health promotion interventions that are relevant to women in the MT seem not only desirable, but a necessity.

In summary, published observational data suggest that women undergoing the MT are at increased risk of clinically significant depressive symptoms and highlight numerous health and behavioural factors that might potentially mediate such increased risk. We have designed a health promotion intervention that seeks to address these risk factors systematically and to enhance behaviours associated with good mental health outcomes. More specifically, this study will aim to address the following:

1. determine the efficacy of a telephone-medicated health promotion intervention, compared with usual care, in decreasing the 12-month incidence of clinically significant symptoms of depression in women undergoing the menopausal transition who do not have clinically significant symptoms of depression or mental disorders at the time of entry into the study,
2. determine the efficacy of a telephone-mediated health promotion intervention in decreasing the 12-month incidence of major depressive episodes in women undergoing the menopausal transition, compared to usual care,
3. measure the effect of a telephone-mediated health promotion intervention in decreasing the severity of depressive symptoms over 12 months compared to usual care,
4. ascertain the prevalence of clinically significant symptoms of depression among community-dwelling Western Australian women undergoing the menopausal transition.

## Methods/Design

### Trial design

The Healthy Transition Trial (HTT) is a randomised, parallel, controlled trial of a telephone-driven health promotion intervention designed to prevent the onset of clinically significant symptoms of depression during the MT. The allocation ratio will be 1:1.

### Participants

We will use the Australian Electoral Commission roll to obtain a random extraction of 40,000 women aged 45 to 55 years living in the Perth metropolitan area (enrolment to vote is mandatory for all adults in Australia). They will be contacted by post to confirm their perimenopausal status (menopausal transition) and to assess the prevalence of clinically significant symptoms of depression (Patient Health Questionnaire, PHQ-9 score ≥15). They will be asked to return their screening questionnaire alongside the signed consent form, and those with PHQ-9 < 15 will be invited to join the trial. This screening questionnaire will take approximately 30 minutes to complete and will collect the following information:

1. demographics including date and place of birth, age, marital status, number of children, educational attainment, living arrangements;
2. regularity of menstrual cycles to establish menopausal status;
3. history of life-limiting medical illnesses;
4. presence of significant hearing or visual impairment;
5. current and past common medical conditions, including depressive and anxiety disorders;
6. alcohol use, as measured by the Alcohol Use Disorders Identification Test (AUDIT, details below);
7. history of gynaecological surgery and medical procedures (for example, contraceptive implants) that may affect the assignment of menopausal status;
8. prescription and non-prescription medications (including hormone therapy);
9. depressive and anxiety symptoms, as rated by the Hospital Anxiety and Depression Scale (HADS, details below);
10. past history of schizophrenia, schizoaffective, delusional or bipolar disorder; and
11. general practitioner’s (GP) details and permission for the study team to liaise with GP.

For the trial, we will include women who are:

1. aged 45 to 55 years;
2. experiencing no, or sub-threshold depressive symptoms (PHQ-9 < 15);
3. experiencing recent (last 5 years) onset of irregular menstrual cycles (≥7 days difference in length of consecutive cycles) OR ≥2 skipped cycles and at least one interval of amenorrhea of 60 or more days; and
4. amenorrhoeic for <12 months.

Volunteers will be excluded from participating for the following reasons:

1. they disclose a history of gynaecological surgery that compromises the ability to assign menopausal status (that is, hysterectomy, bilateral oophorectomy, or endometrial ablation);
2. they report an illness that may impact upon 12-month survival (for example, metastatic cancer);
3. they receive medical treatment or undergo medical procedures that hinder ability to assign menopausal status (hormone therapy, hormonal intrauterine devices, contraceptive implants, patches or medications);
4. there is evidence of clinically significant depressive symptoms (PHQ 9 ≥ 15) or major depressive episode at the time of assessment;
5. there is evidence of concurrent alcohol abuse or dependency (AUDIT ≥15);
6. they report past or current history of schizophrenia, delusional, schizoaffective or bipolar disorder;
7. there is evidence of significant hearing impairment that may compromise telephone communication;
8. they are not fluent in written or spoken English;
9. they plan to leave Western Australia within the following 12 months;
10. they report active suicidal ideation;
11. they do not have an active GP or do not consent to the research team liaising with their GP throughout the course of the study; or
12. they fail to provide written informed consent.

### Ethics

The Research Ethics and Biosafety Office of University of Western Australia approved the research protocol and procedures of the study (RA/4/1/4790). This trial complies with the principles of the Declaration of Helsinki and all participants will be required to provide written informed consent.

### Intervention

The intervention will consist of eight 30- to 45-minute telephone sessions delivered by a qualified clinical psychologist trained to work in health promotion. These sessions will aim to accomplish the following:

1. promote a positive approach to the menopausal transition through the provision of quality information and building of skills and capacity to manage identified issues;
2. offer verbal and written access to evidence-based information about the MT and its management and, where relevant, depression, anxiety, and about the association between depression and other health issues, such as diabetes, hypertension, lipid abnormalities, and other cardiovascular factors;
3. promote changes in hazardous lifestyle practices associated with increased risk of depression and associated with poor health outcomes (physical inactivity, tobacco smoking, excessive alcohol consumption, and poor nutrition);
4. optimise the management of chronic medical conditions and of depressive symptoms;
5. empower participants by offering access to relevant educational material/health resources and by helping them improve problem-solving techniques and rescheduling of activities; and
6. promote symptom monitoring and active surveillance of depressive symptoms.

We will post written educational material about the MT, depression and healthy lifestyle practices to intervention participants before their first session. Sessions will take place within 2 weeks of the baseline assessment and again after 2, 4, 8, 12 and 26 weeks [30]. The health promotion officer will use motivational interviewing and problem-solving techniques to address issues that are identified as relevant by the participant (for example, hot flushes or physical inactivity). The intervention will be tailored to the individual needs of each participant, based on the stages of change model and ‘patient-centred’ approach to care [31]. It will also allow for two extra sessions (total equal eight) to be booked if the health promotion officer and the participant identify issues that were not addressed to their satisfaction during the six structured sessions. If no risks or concerns are identified, the intervention will emphasise education. The structure of the sessions described below will be individualised and the content will be delivered according to needs. For example, if significant anxiety or depression is identified these will be dealt with in earlier sessions. The sessions will cover the following areas:

1. During the first session participants will be encouraged to describe their views/experiences of the MT, concerns or troublesome experiences (for example, hot flushes/night sweats, sleep disturbance, loss of social role, prevalent morbidities, change in body composition, symptoms of anxiety/depression, *etcetera*). The Health Coach (HC) will provide information about the physiology of the MT and, if relevant, anxiety and depression. HC will describe common experiences during MT and strategies used to manage undesirable/unpleasant signs/symptoms. Finally, participants, with the support of the HC, will be encouraged to prepare a hierarchical list of troublesome experiences and discuss ways to modify them (for example, lifestyle changes, relaxation techniques). HC and participant will then agree on homework activities. If no major issues are identified, the focus will be on education about the MT;
2. The second session will start with review of homework and feedback. HC will then explain the principles of problem solving using examples from session 1 and from feedback, if available. The participants will be encouraged to prepare list of problems they are currently facing that can and cannot be changed, and will then be asked to select most relevant problem that can be changed, list all possible solutions, and choose the most appropriate one. The session will be completed with the HC encouraging participants to prepare diary of problems and strategies used to mitigate them during the following week.
3. Session three will start with a review of homework and discussion of what did and did not work, as well as ways to get around barriers. HC will then highlight key aspects of a healthy lifestyle, with particular emphasis on physical activity, nutrition, tobacco smoking and use of alcohol. Participants will be encouraged to review how their current lifestyle practices conform to existing guidelines, and then highlight practices that they would like to change. The HC will ask them to prepare hierarchical list of possible changes and strategies to be used, and to consider obstacles, facilitators and outcomes using problem solving techniques.
4. Session four will start with a review of homework and discussion of what did and did not work, as well as ways to get around barriers using problem solving. Participants will then be asked to review their current health problems, how those should be managed and how they are being managed in practice. Difficulties complying with medical advice will be listed, as will be strategies that could optimise health care. The latter will follow the principles of problem solving. Participants will be encouraged to prepare a list of how their health and well-being may be optimised, as well as strategies that could be used to achieve and sustain those changes.
5. Session five will start with a review of homework and discussion of what did and did not work, as well as ways to get around barriers using problem solving. Participants will then be encouraged to describe their understanding of what depression is, as well as its key symptoms and treatment options. The HC will then discuss with participants how to identify the onset of clinically significant depressive symptoms and strategies to respond to their presence appropriately (where/how/why to seek help, who to contact). During the subsequent week, participants will be encouraged to prepare and maintain diary of MT symptoms, lifestyle practices and management of current medical problems as well as active monitoring of depression, including obstacles, facilitators and strategies to overcome them.
6. During the sixth session, the HC will review homework activities and will encourage discussion of what did/not work and ways of getting around obstacles using problem solving. The HC will subsequently provide information about the association between lifestyle practices, MT symptoms, chronic medical diseases and depression, and will review strategies to optimise good MT and minimise the risk of depression. The session will be concluded with a summary of key-points covered during the previous sessions and organisation of up to two additional sessions to address lingering problems or concerns.

Participants who are assigned to usual care (control group) will not have access to the health promotion officer but will take part in the collection of endpoints in the same way as women allocated to the intervention. As for women in the intervention group, clinically significant findings will be communicated to treating GPs.

### Outcomes and study measures

The onset of clinically significant symptoms of depression or of a major depressive episode over 12 months represents the primary endpoint of interest of this trial. We will use the Patient Health Questionnaire (PHQ-9) to establish the presence of clinically significant symptoms of depression. The PHQ-9 is the self-administered depression module of the PRIME-MD diagnostic instrument for common mental disorders. It consists of nine questions about how often the respondent has been bothered by depressive symptoms during the past two weeks, and each item can be scored 0 (not at all), 1 (several days), 2 (a week or more) or 3 (nearly every day). The nine items are as follow: (a) decreased interest or pleasure, (b) low mood, (c) sleep disturbance, (d) lack of energy, (e) disturbed appetite, (f) feelings of failure or guilt, (g) poor concentration, (h) psychomotor disturbance, and (i) suicidal thoughts. The scale has well-established psychometric properties and face-validity, with scores of 15 or greater indicating the presence of clinically significant symptoms of depression that warrant treatment [32]. Endpoints will be collected at 8, 26 and 52 weeks. Another primary endpoint of interest is the onset of a DSM-IV-TR major depressive episode during follow-up, as ascertained by the Mini-Neuropsychiatric Interview (MINI) 8, 26 and 52 weeks after the baseline assessment [33]. The MINI can take between 15 and 45 minutes to complete and it will allow us to investigate the presence of depressive episodes that may have occurred between assessments [34]. Only women who score 15 or more on the PHQ-9 will undergo assessment with the MINI.

Secondary outcomes of interest of this trial include changes in the severity of depressive and anxiety symptoms as measured by the Hospital Anxiety and Depression Scale (HADS) [35], health-related quality of life as measured by the 12-item Short-Form Health Survey (SF-12) [36], severity of menopausal symptoms as measured by the Menopause Rating Scale (MRS) [37], changes in lifestyle as measured by the amount and frequency of alcohol consumed during a typical week, time spent in vigorous and non-vigorous physical activities during a typical week, the Food Frequency Questionnaire [29, 38], weight and height, and use of psychotropic medications. We will also ask participants to disclose the following sociodemographic and clinical data: age, place of birth, education attainment, marital status, number of children and medical history, including information about past diagnosis or treatment for hypertension, diabetes, arthritis, asthma or other chronic respiratory diseases, coronary heart disease, stroke, neurodegenerative diseases (such as Parkinson’s disease), depression and anxiety disorders. Table 1 summarises the assessment schedule of the trial.

**Table 1:** **List and timeline of assessments for the Healthy Transition Trial (HTT)**

	Screening	Baseline	2 months	6 months	12 months
Sociodemographic information	YES	-	-	-	-
Patient Health Questionnaire (PHQ-9)	YES	YES	YES	YES	YES
Five-year menstrual history	YES	YES	YES	YES	YES
Gynaecological history	YES	YES	YES	YES	YES
Past history of severe mental disorder	YES	-	-	-	-
Severe medical illness (for example, metastatic cancer)	YES	-	-	-	-
Medical morbidities checklist	YES	YES	YES	YES	YES
Alcohol Use Disorders Identification Test	YES	YES	YES	YES	YES
Self-reported hearing or visual impairment	YES	-	-	-	-
Self-reported English fluency	YES	-	-	-	-
Contact details for general practitioner	YES	-	-	-	-
Hospital Anxiety and Depression Scale (HADS)	-	YES	YES	YES	YES
Mini-Neuropsychiatric Interview	-	YES	YES	YES	YES
Short-Form Health Survey, SF-12	-	YES	YES	YES	YES
Menopause Rating Scale (MRS)	-	YES	YES	YES	YES
Food Frequency Questionnaire	-	YES	YES	YES	YES
Prescription and non-prescription medications	-	YES	YES	YES	YES
Self-reported weight (Kg) and height (cm)	-	YES	YES	YES	YES
Smoking, alcohol and physical activity	-	YES	YES	YES	YES

### Sample size

Data from the Australian National Survey suggest that the prevalence of clinically significant depression is 14% around the MT and about 6% immediately before the MT or after the menopause [5]. An effective intervention should decrease the prevalence of depression amongst these women from 14% to 6%, which would represent an absolute risk reduction of 8%. The expected number of women that we will need to treat to prevent one person developing depression is 13. A sample size of 300 women (1:1 allocation) will give the study 80% power to declare such a difference as statistically significant (alpha = 5%). These numbers take into account a loss of 40 women during the course of the study.

### Randomisation and blinding

People with sub-syndromal symptoms of depression are at greater risk of developing a full-blown depressive episode during the subsequent year than those free of such symptoms [39]. In order to minimise the risk of imbalance, we will stratify the randomisation procedure of women deemed eligible to join the study according to the presence or absence of subsyndromal symptoms of depression, which will be defined by PHQ-9 scores between 5 and 14, inclusive. Participants in each of these two strata will be randomly assigned to the intervention or usual care according to a list of random numbers generated by computer in blocks of 12 or 24. As a consequence of the design of this study, participants and the Health Promotion Officer will not be blind to group allocation, but staff responsible for the administration and coding of the MINI will be blind to it. Both participants and staff will be instructed not to discuss any aspects of the intervention during assessment with the MINI. We are also mindful of the fact that history of past depression is a risk factor for the development of subsequent depressive episodes. Because of the randomisation, history of past depression should not bias the results of the trial. Nonetheless, we will monitor the study for possible imbalances and will adjust the analyses of outcomes, if necessary.

### Statistical methods

Data analysis will be blind to group assignment. We will use descriptive statistics to summarise study measures and will compare their distribution at the baseline assessment for the intervention and control groups using t-tests, Mann-Whitney tests and Pearson’s chi-square statistic. Analyses of outcomes will be intention-to-treat (ITT) and will be ascertained using xtlogit models for binary outcomes (for example, developed a depressive episode during follow up: yes/no. Effect expressed as odds ratio and respective 95% confidence limits) and multilevel mixed-effects linear regression (xtmixed models) to analyse changes in scale scores over time (for example, changes in HADS scores - effect estimate expressed as mean change of score from baseline). We will also use contingency tables to calculate the number needed to treat (NNT) for one person to benefit from the intervention, taking into account the number of participants who may be lost during follow up. The NNT is the reverse of the absolute risk reduction. Alpha will be set at 5% and all statistical tests will be two-tailed. Data from this trial will determine if the intervention causes benefit, harm or neither.

## Discussion

Our strategy to recruit participants using the electoral roll aims to increase the external validity of the results of this study and offers the additional flexibility of increasing the number of people approached, if necessary. We have an established record of success for recruiting participants into large community-based trials and of collaborative work with primary care physicians, such as the Health In Men Study [40] and the Depression and Early Prevention of Suicide in General Practice (DEPS-GP) project [26]. We are therefore confident that our recruitment strategy will yield the required number of participants.

We have also designed an intervention that takes into account the reality of health care delivery in Australia, so that the study activities can later be incorporated into normal clinical practice through existing Medicare Benefits Schedule. Sustainability requires evidence of efficacy and of cost-effectiveness to lead to a commitment to long-term implementation. This study aims to establish evidence of efficacy. Furthermore, we broadly estimate that the total cost of the intervention per participant will be about A$412: $50 x 8 for health coaching + $2 × 6 for correspondence. We anticipate that 13 women will need to be treated for one to benefit, which would result in a total cost of about A$5,500 to avoid one case of depression. The estimated annual cost of depression per patient in Australia is approximately A$17,000 (considering an extremely conservative 30% increase in costs since the publication of Australian estimates in 2003) [41]. This would result in a minimum net gain of $11,500 per year for every 13 women treated (savings in other health areas have not been taken into account, so savings could be even greater). These preliminary calculations indicate that the intervention will be cost-effective if the expected benefits are confirmed.

Current evidence shows that depressive symptoms and disorders are leading causes of disability worldwide that are relatively common during the MT [42]. Targeting at risk populations with preventive strategies is a cost-effective approach to avoid further morbidity and mortality [43]. No previous studies have targeted prevention in women at risk during the MT. This study is innovative in proposing the use of a sound multifaceted health promotion strategy to prevent depression during the MT. The intervention will seek to empower women in their decisions about beneficial changes in their lives. Our approach is patient-centred and aims to synergistically enhance the benefits of treatments that participants require, so that fewer women undergoing the MT will experience clinically significant symptoms of depression (minimisation of risk). Positive findings from this trial will provide the required evidence-base for setting up future simple and cost-effective interventions to assist women move successfully through the MT.

## Trial status

The HTT is currently recruiting participants.

## Abbreviations

AUDIT: Alcohol Use Disorders Identification Test
GP: general practitioner
HADS: Hospital Anxiety and Depression Scale
HC: Health Coach
HTT: Healthy Transition Trial
ITT: intention to treat
MINI: mini-neuropsychiatric inventory
MRS: Menopause Rating Scale
MT: menopausal transition
PHQ-9: Patient Health Questionnaire
PS: problem solving
NNT: number needed to treat
SF-12: Short Form Health Survey.
